# Supplementary material for: Are carbon emissions trading and green financial instruments synergistic? -Comprehensive quantitative research based on content analysis
Source: PLoS One. 2024 Mar 7;19(3):e0298601. doi: 10.1371/journal.pone.0298601 (PMC10919601; doi:10.1371/journal.pone.0298601)
Supplement: S1 Data — (ZIP) [file pone.0298601.s001.zip › Data/地级市Z_F工作报告.zip!/╜¡╦╒/╒≥╜¡╩╨/└┤╫╘╣1⁄2╓┌║┼í╛┬φ┐╦╩2╛▌═°í┐_2012.docx]

各位代表：

　　现在，我代表镇江市人民政府向大会作工作报告，请予审议，并请市政协委员和其他列席人员提出意见。

　　2011年工作回顾

　　过去的一年，在省委、省政府和市委的正确领导下，全市上下深入贯彻落实科学发展观，坚持“六个注重”、推进“八项工程”，圆满完成市六届人大四次会议确定的各项目标任务，实现了“十二五”发展良好开局，顺利开启了基本现代化新征程。预计全市实现地区生产总值2300亿元，比上年增长12%；地方财政一般预算收入181.9亿元，增长31.7%；固定资产投资1225亿元，增长22.5%；社会消费品零售总额660亿元，增长18%；实际利用外资18亿美元，增长11.5%；进出口总额突破100亿美元，增长22.7%；城市居民人均可支配收入26200元，增长13.5%；农民人均纯收入12560元，增长15.5%；单位GDP能耗下降3.7%，主要污染物减排完成省下达任务。

　　一、经济结构持续优化

　　现代服务业量质齐升。增加值占GDP比重达40.8%。3个城市综合体建成运营，“一主五副”六大商圈初具形态。“云神工程”全面启动。软件业实现营业收入160亿元、增长55.3%，新增软件企业100家；服务外包执行额3.2亿美元，增长103.8%。国家级智慧旅游服务中心落户镇江，11家高星级饭店项目加快推进，旅游总收入超400亿元。文化产业完成投资59.5亿元、增长65.4%，增加值占GDP比重突破5%。金融服务体系进一步完善，农商行、村镇银行和异地农商行实现辖市全覆盖，新开业11家农村小额贷款公司，新增4家证券营业部、8家创投机构。3家新上市公司募集资金8亿元。应收账款质押和知识产权质押融资走在全省前列。先进制造业加快发展。“千百亿工程”深入推进，五大主导产业实现销售突破4000亿元。五大新兴产业完成投资407亿元，增长100.6%；实现销售1640亿元，增长54.6%。创成2家省级特色产业集聚示范区，新增千亿产业、百亿企业各1个。新落户央企投资项目11个，其中新兴产业类占82%。农业现代化提速增效。农业增加值突破100亿元，粮食生产实现“八连增”。新增4家省级现代农业产业园区，率先实现省级园区辖市全覆盖。新增亩均效益万元以上设施农业面积超10万亩。吸引“三资”投入农业51亿元，合作组织入社农户数超10万户。疏浚镇级以上河道48条、村庄河塘618个，水利现代化进程不断推进，改革发展经验在全国推广。

　　二、科技创新成效明显

　　成为国家创新型试点城市，第八次荣获全国科技进步先进市称号，科技进步综合实力列全省第五位。全市科技进步贡献率达55.5%，全社会研发投入占GDP比重达2.2%。高新技术产业产值占比达42.5%，列全省第二位。省级以上科技计划项目立项近500项，获拨款超2.5亿元。成功引进和建设11家省级以上研发、检测机构，新增孵化面积超50万平方米，建成7家省级商务公共服务平台。新“331计划”成效显著，新引进领军团队71个、领军人才263名，新入选国家“千人计划”8名。引进本科以上人才1.2万名，培养高技能人才8000名。

　　三、城乡建设扎实推进

　　全市完成建设投入570亿元。南徐新城行政中心投入使用，档案馆、科技馆建成交付，商务A区、职工文体中心和体育会展中心等主体竣工，金山湖景区调水控水工程全面完成，官塘片区开发全面启动。市区新改扩建道路24条，建成公交首末站场18个，住宅小区实现物业管理全覆盖，大市口地下人防工程投入使用。金山水厂扩建工程完工，区域供水普及率达98.7%，创成国家节水型城市。数字镇江地理空间框架建设项目获批，成为国家级数字城市推广建设市。深入推进“大城管”建设，建成区道路实现保洁一体化，生活垃圾焚烧发电厂建成使用。交通基础设施投入99亿元，创历史新高。338省道市区段改造完成，大路飞机临时起降点立项获批。镇江港口货物吞吐量达到1.15亿吨。丹阳、句容和扬中市中心城区建设步伐加快，丹徒新城与主城全面对接，京口、润州城市经济承载能力进一步增强，镇江新区“万顷良田”工程得到国土资源部充分认可。全市村镇“三新”建设快速推进，第一批87个村庄环境整治全面启动。

　　四、生态建设力度加大

　　顺利通过国家环保模范城市复核，成为全国第一个按新标准通过复核的省辖市。国家生态市建设顺利推进，三个辖市全部通过国家级技术评估。全市生态镇占比达87.8%、生态村占比达65%，林木覆盖率达23.9%，城镇绿化覆盖率达41.3%。获国家森林城市创建立项。“青山绿水”行动继续推进，市区9条河流整治达标，新建污水管网126公里，基本实现污水处理设施全覆盖。建成在线监控网络，实现重点污染源数字化监管。集中整治环境突出问题成效明显。

　　五、社会建设创新发展

　　民生持续改善。公共财政投向民生124.5亿元、增长37.6%，高于一般预算支出增幅近7个百分点。新增城镇就业9.8万人，转移农村劳动力3.6万人，高校毕业生就业率95%，连续72个月保持城镇“零就业”家庭动态为零，城镇登记失业率控制在2.35%以内。创业型城市中期评估综合排名全国第九。医疗、失业、工伤、生育等社会保险市级统筹稳步推进。率先在全省实施失业人员医保新政策，被征地农民社会保障工作取得较好进展。企业职工最低工资标准达1140元、增长18.8%，企业工资指导线提高15%。新建各类保障性住房1.8万套（间）、100.6万平方米，超过前三年总和。市区改扩建16个菜市场，建成35家“菜篮子”平价商店。切实保障困难群体基本生活，建立低保金自然增长机制，发放困难群体物价补贴1861万元，全面消除年收入低于3500元的农村贫困人口。社会事业蓬勃发展。国家学前教育体制改革试点深入推进，东方职校等顺利迁建，教育综合能力继续提升。医改镇江模式和社区卫生“3+X”团队服务在全国产生较大影响。成功举办“长江草莓音乐节”、“文化嘉年华”和“欢乐家园”等300多场文化活动。切实加强文化遗产保护，复建文宗阁、重修北固楼。文化发展绩效考核列全省第三，成为全国文化体制改革先进地区。网络发言人、网上居委会和网上派出所等做法取得实效，互联网应用与管理经验在全国推广。全民健身计划深入推进，率先开通“10分钟体育健身圈”网络服务平台，实现全国残运会金牌零的突破。人口计生工作扎实开展，出生缺陷干预取得明显成效。社会保持和谐稳定。全面启动“六五”普法，扎实推进“平安镇江”、“法治镇江”建设。积极开展中心社区试点，形成了“多网叠加”的社区管理服务模式。市人民来访接待中心正式运行，信访工作取得新成效。成功六创全国双拥模范城。国防教育、人民武装、国家安全工作取得新成绩，丹阳成为全军军民融合示范区。援藏援疆工作有序推进，首批援疆项目完成率全省第一。安全生产、食品药品和产品质量安全工作扎实有效。慈善助医被评为省慈善最具影响力项目。外事、侨务、对台事务、统计、民族宗教、地方志、档案、防震、老龄、科普、妇女儿童和关心下一代等事业继续加强。

　　六、自身建设不断加强

　　自觉接受市人大及其常委会的法律监督、工作监督，主动接受市政协的民主监督，认真听取各民主党派、工商联、无党派人士和各人民团体的意见。办理人大代表建议260件、政协提案456件，满意和基本满意率达100%。深入开展法治政府建设，制订邀请公民列席市政府常务会议实施办法，市政府常务会议视频在网上播出，政府决策的科学化、民主化程度不断提高。行政权力网上公开透明运行近15万件，“12345”服务热线受理市民来电近20万次。制订网上行政服务规范国家标准，圆满完成两级政府机构改革评估，构建全市政务系统纵向联动六大机制。认真开展预算执行、民生资金和重大项目等绩效审计。扎实推进公务用车专项治理。“中国镇江”政府门户网站绩效评估列全国地级市第11位。无纸化办公走在全省政府机关前列。

　　成绩来之不易，凝聚着全市人民的智慧和辛劳。在此，我谨代表市人民政府，向所有为镇江经济社会发展作出贡献的人们，向所有关心支持镇江发展的海内外朋友，表示衷心的感谢！

　　各位代表，我们也清醒地认识到，工作中还存在一些问题：经济结构性矛盾仍较突出，破解各种要素制约的难度很大，城乡建设、发展和管理的水平有待进一步提升，社会建设中的基础性工作还需要进一步加强等。对此，我们将高度重视、认真整改，努力把工作做得更好。

　　2012年主要任务

　　2012年是继续推进现代化建设的关键一年，面对复杂多变的发展环境，我们将坚持稳中求进，保持发展态势平稳、物价总水平基本稳定和社会大局稳定，在转变经济发展方式、深化改革开放和改善民生上奋力进取，扎实推进现代化进程，不断向上攀登，不负人民厚望！

　　今年政府工作的总体要求是：以科学发展观为指导，全面贯彻中央和省一系列重要会议精神，认真落实市委六届二次全会的决策部署，坚定信心、稳中求进，强化人才引领，加快结构调整，推动科技创新，深化改革开放，加强节能减排，持续改善民生，促进社会和谐，以全面推进现代化进程的优异成绩迎接党的十八大胜利召开。

　　根据省定基本现代化指标体系，今年全市经济社会发展的预期目标为：GDP增长11%；地方财政一般预算收入增长18%；固定资产投资增长18%，其中新兴产业投资增长60%、现代服务业投资增长25%以上；实际利用外资增长8%；进出口总额增长10%，其中出口增长10%；社会消费品零售总额增长17%；服务业增加值占比达42%；全社会研发投入占比达2.4%；高新技术产业产值占比达45%；城市化水平达到64%；城市居民人均可支配收入增长13%，农民人均纯收入增长14%；城镇登记失业率控制在3.5%以内；居民消费价格总水平涨幅控制在4%左右；单位GDP能耗下降率和主要污染物排放削减率完成省下达任务；人口自然增长率低于3‰。

　　工作中，我们将全力加速五个现代化进程：

　　一、提升产业发展现代化水平

　　大力推进创新驱动。顺应苏南地区整体发展战略规划的要求，继续推进国家创新型城市试点工作，大力发展创新型经济，力争完成高新技术产业产值2600亿元，新引进与建设省级以上研发、检测机构10家，科技进步贡献率达58%。切实加强企业和企业家队伍建设，加快培育拥有自主知识产权的创新型企业与名牌产品，更大力度引导创新要素向企业集聚。继续加快人才国际化进程，力争新增国家“千人计划”8人、入选省“双创”计划超40名、省创新团队2个以上；市级科技人才专项资金计划安排1.43亿元、比上年增长42.3%，积极探索财政投入向基金滚动式方向发展；高度重视人才引进后的服务、培养和成长，不断提高项目“成活率”，力争使人才工作继续走在全省前列。全力推进金融创新，通过私募、风险投资等各种途径，助推企业转型升级，确保通过国家知识产权质押融资试点市验收，争创国家知识产权投融资示范市。

　　加快发展现代服务业。抢抓成为“三网融合”试点城市机遇，加快推进“云神工程”，建设智慧城市。大力引进国内外知名电子商务企业，鼓励有条件的企业发展网上连锁经营。建成服务外包载体超200万平方米，争创省级公共服务平台和服务外包特色产业园区。软件业营业收入突破200亿元。建成7个城市综合体。力争再引进2家股份制银行，新增3家上市公司。引导和支持房地产行业平稳发展。新增3个省级以上现代物流载体。大力推进智慧旅游示范城市建设，构建国家智慧旅游公共支撑体系。改革“三山”风景区管理体制和运营机制，力争创成国家5A级旅游景区。

　　持续推进工业优化升级。继续推动新兴产业爆发式增长，力争五大新兴产业投入650亿元、销售2200亿元，争创2家省级以上新兴产业特色基地和新型工业化示范基地。继续推进“千百亿工程”，全面落实各项扶持政策，培育规模企业，新增1家百亿企业。加快“两化融合”步伐，推动传统产业改造升级，确保工业投资完成850亿元、销售突破6000亿元。

　　积极发展现代农业。争创2个省级现代农业产业园区和连片万亩的农业示范园区，新增亩均效益万元以上设施农业面积10万亩，确保高效设施农业面积比重达到12.8%、适度规模经营比重达到60%。提升农村合作组织的运作管理水平，力争农户参加的比重达48%。继续加强农田水利建设，加大水利重点工程建设力度，加快水利现代化进程。认真落实农民收入倍增计划，确保全市薄弱村集体经营性年收入超过50万元、低收入农户人均纯收入超过4000元。

　　二、提升城乡发展现代化水平

　　规划适度超前。以建设山水花园城市为目标，进一步提高规划的前瞻性和现代意识，从群众最期盼、发展最急需、土地最集约、操作最可行的角度，科学编制和组织项目，加快推进城市总体规划修编，完善建成区控制性详规，全方位提升城乡规划编制水平，着力解决好群众反映强烈的出行畅通、生活便利、环境舒适等问题。

　　城建打造亮点。力争全年城建投资完成550亿元。北部滨水区重点启动西津湾、中华路和征润洲片区开发，规划建设长江路金融街区，优化长江路沿线布局，打造完整的滨水风光带；开通金山湖水上游览线路，新建佛祖舍利展示馆，实施焦东湿地景观保护工程。南徐新城核心区完成基础设施建设，商务A区全面建成，商务B区全面开工、部分封顶，建成市体育会展中心，开工建设高铁商务区。官塘新城加快推进路网建设，全面启动电力及景观水系改造，3-5个现代服务业项目落地开工。中心城区加快建设大市口A、B地块等项目，开工建设“水游城”、国贸中心广场等项目。完成主城区老铁路范围内所有“城中村”改造。完成城市三大出入口和古运河中段综合整治，打造学府路、朱方路等一批景观特色街。建成智能公交系统，新建20个公交站场，新增公交线路25条，城市、镇村公交覆盖率均达100%。加快构建大交通格局，基本建成泰州大桥镇江接线主体工程，开工建设镇丹高速和镇江—扬中快速直达通道，实施沪宁高速镇江支线收费站南移，推进338省道丹阳段拓宽改造，形成覆盖全市的快速骨架路网。完成镇江轨道交通线网规划编制。加快推进苏南运河“四改三”工程。全面开工建设大港四期工程，实现港口集装箱业务资源整合。

　　管理精致长效。进一步完善城市管理体制和工作机制，强化属地管理责任，充分发挥街道、社区的基层基础作用。以精致管理为目标，推进管理的网格化、标准化、精细化和常态化，实行实时监控、动态监管，有效解决违法建设、违章占道、店面整治、毁绿种菜和犬类管理等突出问题，建成省优秀管理城市。着力缓解主城区交通拥堵问题，搬迁南门大街夜市，改造和拆除青年广场、梦溪广场，建设2个立体停车库，切实加强渣土车、电动车管理。

　　乡村展示新貌。加快推进城乡规划、产业发展、要素配置、基础设施和公共服务一体化，做到“一村一规划、一村一张图”，打造各具特色的美丽乡村。全面完成主要交通干线沿线和省级以上风景名胜区周边村庄的环境整治，建成40个省级“康居示范村”，完成村庄环境整治总数量的45%。全面完成“三新”建设三年行动目标任务。加快建设农村现代商品流通服务网络。

　　三、提升生态建设现代化水平

　　推进生态文明建设。大力实施生态文明建设工程，深入开展突出问题集中整治。加大国家级生态市建设力度，确保通过环保部考核验收。扎实推进国家森林城市创建，完成合山、黄鹤山、宝塔山等山体整治，保护南山等山体资源，实施山体林相改造，林木覆盖率达到24.8%，城镇绿化覆盖率达到42%。切实加强农村工业和生活污染治理，积极推进植保专业化，有效防治种植、畜禽、水产养殖等面源污染。

　　优化区域环境质量。加强太湖流域水污染防治，落实环境资源区域补偿制度，对完不成治理任务的乡镇实施挂牌督办、区域限批，确保考核断面水质全面达标。继续做好长江水污染防治工作。增强农村污水处理能力，确保乡镇以上污水处理厂全面规范运行。加大集中式饮用水源保护力度，区域集中供水普及率提高至98.8%。大力实施蓝天工程，开展PM2.5等指标监测分析，实施黄标车区域限行，加强扬尘、工业废气等污染综合治理，城市噪声达到功能区标准。推进7个省级以上园区的生态工业园区创建，开展4个化工园区环境保护集中整治行动。

　　完成节能减排任务。启动新一轮三年淘汰落后产能行动计划，实行单位GDP能耗下降和地区能源消费总量“双重”控制，加强投资项目节能评估审查和节能竣工验收。狠抓重点用能企业监管，坚决实施差别电价、惩罚性电价等措施。加快发展循环经济和低碳经济，推进公共机构节能，全社会节能工作通过国家考核。强力推进污水处理设施配套建设，确保污水管网建成运行，全面完成节能减排约束性指标。

　　四、提升文化事业现代化水平

　　积极发展公益文化。深入实施文化建设工程，积极创建省公共文化示范区。建立文化市场综合信息管理系统，继续提升互联网的建设、运用与管理水平。深化文化惠民系列活动，进一步加强农村文化工程建设，打造城市“15分钟文化圈”和农村“十里文化圈”。

　　加快发展文化产业。突出文化创意、影视演艺、旅游文化、现代传媒和数字出版印刷等产业发展，精心打造西津渡民国文化旅游和古渡文化旅游项目，建成丹徒米芾书法公园、丹阳天地石刻园等20个重点文化产业项目，启动市文化艺术中心建设，大力引进核心层、科技型、品牌响的文化企业和产业项目，引进和培养高层次文化人才，力争文化产业增加值占GDP比重达6%。

　　不断扩大文化影响力。繁荣文艺创作，开展丰富多彩的群众文化活动，培育基层特色文化，提高市民的文化自觉和自信。进一步打造“大爱镇江”、“长江音乐节”等品牌。充分利用文化遗产保护成果，举办全国碑帖、古琴等研讨活动，扩大国家历史文化名城的影响。

　　五、提升社会建设现代化水平

　　提高群众生活水平。继续推进创业型城市建设，新增创业5000人，创业带动就业2.5万人，力争新增城镇就业6.5万人、转移农村劳动力2万人。不断扩大创业投资领域，增加居民经营性和财产性收入。全面推行企业工资协商机制，力争企业职工最低工资标准增长15%左右。进一步加大投入，推进社会保障两个“全覆盖”、“五险合一”和被征地农民社会保障等工作，确保城镇职工基本养老保险覆盖率达到98.3%、新型农村养老保险覆盖率达到95%、城乡各类社会医疗保险覆盖率达到96%、失业保险覆盖率达到98%。完成保障性住房建设 200万平方米，努力实现住房困难低收入家庭应保尽保。全力稳定物价，推进平价商店建设，做好动态物价补贴发放工作，保障困难群众基本生活。扩大社会救助覆盖范围，切实加大对低收入群体的帮扶力度。加强养老机构建设。做好市民卡规模化发放工作。实行残疾人免费乘坐公交车。

　　提升公共服务水平。深入推进教育现代化，继续深化国家学前教育体制改革试点，加快推进中小学布局优化调整，主要劳动年龄人口平均受教育年限提高到9.7年。全面完成校舍安全工程三年规划，积极推行校车公交化。扎实开展“健康镇江”行动，建成区域卫生信息平台，提高市民健康水平。深入推进医疗集团实体化运作，完善“3+X”团队服务模式，大力提升社区卫生服务水平。推进城乡公共体育设施建设，推动群众体育活动蓬勃开展，进一步做响乒乓、象棋、篮球等品牌。推动计生卫生合作和资源共享，实施人口计生公共服务均等化，确保全市出生缺陷发生率控制在8‰以下。深入开展群众性精神文明创建活动、科普活动和各类培训，提高市民文明素养和社会文明程度，促进人的现代化。

　　促进社会和谐稳定。深入开展和谐社区创建活动，建设10个邻里中心模式的社区服务示范区。继续加强政务诚信、商务诚信、社会诚信和司法公信建设。推进法治镇江建设，抓好“六五”普法，率先实现法治政府建设阶段性目标，力争2个辖市区跨入法治县市区创建工作先进行列，建成全省首批法治城市。坚持“四民主、两公开”，推进农村基层民主法制建设。围绕贯彻主题主线重大战略思想，扎实推进国防动员和后备力量建设。按照“全面融合、全面提升”的新思路，七创全国双拥模范城。深化平安镇江建设，深入开展社会治安整治专项活动，做好流动人口服务管理。落实信访维稳工作领导责任制，坚持领导干部定期接访和下访，深化大调解机制建设，依法处理好信访问题。扎实做好安全生产、食品药品安全和应急保障等工作，严防重特大事故发生。

　　各位代表！现代化建设，呼唤现代服务型政府。我们将进一步加快转变政府职能，深化第六轮行政审批制度改革，推进“两集中、两到位”工作，完成事业单位清理规范，不断扩大市场开放度。我们将进一步加强电子政务和数字政府建设，推动行政权力网上公开透明运行，拓展政府门户网站为民服务功能，大力构建政务微博平台，提供实时公共服务。我们将进一步加强行政监察和审计监督，规范权力运行，健全行政问责机制，切实增强执行力，提升服务效能和服务水平。我们将进一步加强政府廉政建设，坚持不懈地推进反腐倡廉工作，带头勤俭节约，树立全体公务人员勤政、清廉、为民的良好形象。

　　各位代表，天时人事日相催，冬至阳生春又来。新的一年充满希望，新的任务催人奋进。让我们全面贯彻落实科学发展观，在市委的坚强领导下，开拓创新、锐意进取，在率先基本实现现代化的征程上阔步前进！
